# Profiles of Resilience among Children Exposed to Non-Maltreatment Adverse Childhood Experiences

**DOI:** 10.3390/ijerph182010600

**Published:** 2021-10-10

**Authors:** Susan Yoon, Nathan Helsabeck, Xiafei Wang, Jessica Logan, Fei Pei, Sherry Hamby, Natasha Slesnick

**Affiliations:** 1 College of Social Work, The Ohio State University, Columbus, OH 43210, USA; 2 Quantitative Research, Evaluation and Measurement, College of Education and Human Ecology, The Ohio State University, Columbus, OH 43210, USA; helsabeck.1@buckeyemail.osu.edu (N.H.); logan.251@osu.edu (J.L.); 3 School of Social Work, Falk College, Syracuse University, Syracuse, NY 13244, USA; xiwang@syr.edu (X.W.); fpei01@syr.edu (F.P.); 4 Department of Psychology, The University of the South, Sewanee, TN 37383, USA; sherry.hamby@gmail.com; 5 Life Paths Research Center, Sewanee, TN 37375, USA; 6 Department of Human Sciences, College of Education and Human Ecology, The Ohio State University, Columbus, OH 43210, USA; slesnick.5@osu.edu

**Keywords:** resilience, adverse childhood experiences (ACEs), kindergarten, children, latent profile analysis

## Abstract

Considering the high prevalence and negative consequences of non-maltreatment adverse childhood experiences (NM-ACEs), it is critical to understand their impacts on the resilient functioning of young children. This study sought to examine heterogeneity in resilience among first-grade children who were exposed to NM-ACEs during kindergarten and explore demographic and adversity characteristics that distinguish between resilience profiles. Latent profile analysis (LPA) was conducted on 4929 children drawn from the Early Childhood Longitudinal Study—Kindergarten (ECLS-K). The results of the LPA revealed four distinct resilience profiles: (1) *low cognitive and executive functioning* (4%); (2) *low social and behavioral functioning* (14%); (3) *low average functioning* (31%); and (4) *multi-domain resilience* (51%). Female children and those in families characterized by older maternal age, higher parental education level, household income above 200% federal poverty level, not receiving welfare benefits, and races other than Black were more likely to be in the *multi-domain resilience* profile. The findings highlight heterogeneity in resilience among children exposed to NM-ACEs and point to the need for a comprehensive, multi-domain assessment of child functioning to support optimal resilience development in this population.

## 1. Introduction

Despite its potential importance, the impact of non-maltreatment adverse childhood experiences (NM-ACEs) on child development and resilience has received disproportionately less attention compared to that of child maltreatment. NM-ACEs (often referred to as household dysfunction) have been linked to negative health outcomes during adulthood, such as severe obesity, chronic lung disease, and ischemic heart disease. However, the impact of NM-ACEs on childhood outcomes, particularly positive and resilient functioning, remains unclear. In this paper, resilience is defined as the process of positive adaptation in the midst of adversity [1,2]. Understanding different profiles of resilience among children exposed to NM-ACEs can inform intervention strategies to support vulnerable children and help them achieve positive outcomes. Applying a person-centered analytic approach (i.e., latent profile analysis), the current study contributes to the literature on early childhood development by highlighting children’s resilient development in the context of NM-ACEs, including poverty and parental psychopathology.

### 1.1. Adverse Childhood Experiences

The ground-breaking Adverse Childhood Experiences (ACEs) study has deepened our understanding of the link between childhood adversities and human development [3]. The ACEs study identified ten distinct categories of childhood adversity that encompass child maltreatment (i.e., physical abuse, sexual abuse, emotional abuse, emotional neglect, physical neglect) and stressful family environment (i.e., mental illness, substance abuse, imprisoned household member, parental divorce, domestic violence) [4]. Numerous studies have revealed the detrimental effects of ACEs on individuals’ well-being, including poorer physical and behavioral health, juvenile offending, delinquency and substance use, reduced life opportunities, and shortened life expectancy [3,4,5,6].

Though numerous ACEs studies have extended knowledge on the impacts of ACEs during adulthood, research examining immediate effects of ACEs on early childhood (e.g., kindergarten) is still in its infancy. Kindergarten marks a critical developmental period when children experience rapid brain growth and develop socioemotional and cognitive skills that serve as a foundation for lifelong success [7]. Unfortunately, studies suggest that there is already a high prevalence of ACE exposure among children before school age. Based on a nationally representative birth cohort sample (*n* = 4898) from the Fragile Families and Child Well-being Study, Hunt and colleagues found that 77.4% of the sample experienced at least one ACE by age 5 [8]. A survey relying on a multi-year sample from 2016 to 2018 of the U.S. Census Bureau’s National Survey of Children’s Health suggested that over 25% of children experienced at least one ACE before age 4, with economic difficulty being the most common form of adversity [9].

Given that exposure to ACEs is prevalent among young children and creates a toxic environment where children experience overwhelming stress that hinders healthy brain, cognitive, social, and emotional development [10], more research is warranted to examine the immediate effects of ACEs during childhood. Furthermore, while ample research has examined the effects of child maltreatment (i.e., maltreatment ACEs) on early childhood development [11,12,13], less attention has been paid to NM-ACEs and home environment, such as parental mental health problems, substance use, and household poverty. Several studies have examined the role of home environment, including chaotic homes, on child development and suggested that household chaos (independently or in conjunction with child maltreatment) may contribute to negative developmental outcomes, such as child disruptive behavior [14,15]. Considering that NM-ACEs act as severe risk factors for children by increasing child maltreatment risks and depriving children of a healthy and stimulating developmental environment [16,17], it is critical to understand how NM-ACEs influence positive and resilient functioning in children.

### 1.2. Exposure to NM-ACEs and Resilience

Prior research has found negative effects of non-maltreatment adversities on early childhood development. Studies have suggested a link between caregivers’ mental health problems and children’s behavioral and mood difficulties [18]. For example, parental depression has been shown to hinder parents’ ability to practice positive parenting and form nurturing parent–child relationships, which in turn may lead to decreased behavioral, cognitive, social, and emotional functioning in children [19]. Moreover, parental substance use problems, which often co-occur with child maltreatment, raise concerns for child well-being. Children living with parents with substance use problems are more likely to develop somatic illness and psychiatric disorders [20], exhibit externalizing problems [21], and show poorer school performance [22] and compromised social–emotional outcomes [23]. Furthermore, household poverty—chronic stress that overwhelms children’s physiological response systems—can disrupt children’s self-regulatory skills that are fundamental for social–emotional development [24]. Exposure to poverty is also associated with less efficient brain network organization and the disruption of the brain connectome, especially among girls [25].

However, even in the midst of or after exposure to adversity, some children reveal resilience by showing successful and positive adaptations [2]. A study on premature children living in poverty suggested that those who received responsive, accepting, stimulating, and organized care were distinguished from others by their higher levels of cognitive and social functioning as well as better health and growth development at age three [26]. Research also suggests that despite being exposed to maternal depression, children with mothers holding positive feelings about parenting demonstrate the same level of function as children not being exposed to maternal depression [27]. According to a systematic review on the resilience factors of children of parents with drinking problems, gene differences, self-esteem and self-regulation, positive parenting and secure attachments with parents, positive family climate, the presence of other trustable family members, and social support all contributed to childhood resilience [28].

### 1.3. Theoretical Framework: Resilience Portfolio Model

This study is conceptually guided by the resilience portfolio model [29,30]. The resilience portfolio model centers on using a strengths-based lens to understand the protective factors and processes that promote resilience following exposure to significant challenges and adversities [29]. This framework encourages the investigation of positive aspects of functioning in individuals who have experienced violence and other adversities by paying attention to the roles of various strengths and protective factors across multiple levels and different systems across the social ecology [29]. In other words, the same adverse event (e.g., parental mental health problems) may have different influences on different children depending on additional risks (polyvictimization) or protections (polystrengths) or different systems by which they are surrounded [31]. Therefore, a particular adverse experience does not necessarily lead to the same problematic outcomes for every child, resulting in diverse patterns of resilience in the context of adversity.

### 1.4. Distinct Profiles of Resilience

Resilience should be viewed as a multi-faceted, multi-dimensional construct rather than a unidimensional yes/no phenomenon [32]. Children exposed to adversity may show varying outcomes across different domains of adaptation that include academic, social, and emotional resilience [32]. For example, inner-city youths who are highly appraised by their peers might show poor academic competence [33]. Children of parents with depression may sufficiently cope with everyday stressors but at the same time show vulnerability to psychopathology [34]. The complexity regarding children’s varying developmental outcomes across distinct spheres of resilience calls for a closer examination of the multidimensionality of resilience [35].

Although limited research focuses on resilience following exposure to NM-ACEs, studies on resilience in the context of child maltreatment have shed light on the multidimensionality of resilience [36,37,38]. For instance, Bolger and Patterson (2003) examined four domains of resilience functioning (e.g., peer acceptance, internalizing behavior, externalizing behavior, and academic achievement) for school-age children who experienced child maltreatment [39]. Sattler and Font (2018) defined resilience in kindergarteners involved with child protective services as social resilience, cognitive resilience, and multi-domain resilience (i.e., children who showed both social and cognitive resilience) [40]. Though these studies provided valuable insight in conceptualizing and assessing resilience as a multi-domain construct, they were methodologically limited and could not comprehensively capture heterogeneity (i.e., various patterns) in resilience.

The methodological breakthrough of person-centered analytic approaches [41], such as latent class/profile analysis and cluster analysis, has allowed the examination of distinct and unique patterns of resilience. Using cluster analysis, a study on children exposed to intimate partner violence identified four unique profiles of children’s adjustment: severe adjustment problems, children who were struggling, children with depression only, and resilient children with high competence and low adjustment problems [42]. Employing latent class analysis, Wilson and colleagues (2016) found four distinct profiles of resilience among young black gay and bisexual men: (1) low self-efficacy and adaptive coping; (2) low peer and parental support; (3) high peer support but low father support; and (4) high parental support, self-efficacy, and adaptive coping [43]. While previous studies demonstrated the usefulness of person-centered analysis in identifying various patterns of resilience, this method has not been used with kindergarteners exposed to NM-ACEs to identify resilience profiles in this population. Thus, it remains unclear whether and to what extent different patterns of resilience are shown across multiple domains of functioning (e.g., cognitive, executive functioning, behavioral, social) among young children exposed to NM-ACEs, including poverty. This is an important research gap to address considering the high prevalence of NM-ACEs experienced by young children, including economic difficulty, parental separation, and parental substance use and mental health problems [11]. This study sought to advance the knowledge base on childhood adversity and resilience through the application of a sophisticated modeling strategy (i.e., latent profile analysis) to reveal distinct profiles of resilience in the context of NM-ACEs.

### 1.5. The Current Study

The current study aimed to investigate heterogeneity in resilience and discover various resilience profiles among a nationally representative sample of kindergarteners and first graders, using data from The Early Childhood Longitudinal Study—Kindergarten Class of 2010–11 (ECLS-K:2011). Further, as an exploratory aim, demographic and adversity characteristics were compared across the profiles of resilience. Two research questions guided this study: (1) What different profiles of resilience are displayed by first-grade children who were exposed to NM-ACEs during kindergarten? (2) To what extent do demographic characteristics and NM-ACEs differ across the identified resilience profiles? It was hypothesized that there would be heterogeneous patterns of resilience in children exposed to NM-ACEs during kindergarten. Given that no known research has examined resilience profiles among first-grade children exposed to NM-ACEs, an a priori hypothesis was not formulated regarding the number and shape of the profiles of resilience. Based on prior studies, it was anticipated that older maternal age, higher level of parental educational attainment, female gender, and less exposure to NM-ACEs would be observed among children with profiles of high resilience. The present study makes a unique contribution to the literature by focusing on the impact of NM-ACEs on profiles of early childhood resilience, which is an important yet understudied area that can inform future intervention strategies to promote the healthy development of vulnerable children.

## 2. Materials and Methods

### 2.1. Participants

The current study examined data from the Early Child Longitudinal Study Kindergarten Cohort 2010–2011 (ECLS). The ECLS is a nationally representative study conducted by the National Center for Education Statistics, which collected data from over 18,000 children attending public and private kindergarten in the 2010–2011 school year. Data collected included direct child assessments, teacher assessments of students, parent and teacher interviews, and student and school demographic characteristics. At the time this study was conducted, ECLS data were publicly available through the 5th grade for this cohort of students; at completion, the dataset will include data through the 8th grade. In the current study, we used data collected in the spring of the kindergarten year and spring of the first-grade year. As five of the outcome measures of interest were taken from measures rated by teachers, children with completely missing data on all the teacher-rated measures were excluded from the sample. This reduced the available sample to *n* = 13,475.

Because we were interested in examining resilience following exposure to adversity, we limited our analytic sample to children who have experienced at least one NM-ACE. Following guidance from the available literature [3], we examined variables within the ECLS-K dataset that would help identify children who had experienced adversity early in life. Four NM-ACE variables were selected: poverty, parent depression, parent emotional or substance use problems, and whether a family had received welfare benefits in the past 12 months. It is important to note that although measures referred to kindergarten year, it is likely that some of these adversities (such as poverty) had been present before that for some children.

Our final analytic sample (*n* = 4929) included children who experienced at least one of the four NM-ACE indicator variables and had valid responses for all four variables. We excluded cases that did not have valid responses to any of these four variables. Children in the analytic sample were less likely to be White, had caregivers with younger age and lower educational level, and scored lower on all outcome assessments (resilience indicators) compared to children who were excluded from the study (i.e., non-NM-ACES). There were no other significant differences between the two groups.

Sample descriptive statistics are provided in Table 1. The analytic sample was 50% female with a mean age of 7.2 years in the spring of first grade. Further, the analytic sample was 44% White, 12.3% Black, 32% Hispanic, and 11% coded as “Other” but inclusive of children identified as Asian, Native Hawaiian/Pacific Islander, American Indian/Alaska Native, and two or more races. The mean age for primary caregivers in this sample was 35, with 18.4% holding less than a high school degree or equivalent. Finally, 74% of students in this sample lived in a household that was below 200% of the poverty threshold. Such a high percentage of children living in poverty is likely related to poverty status being one of the possible inclusion criteria for being selected into the analytic sample.

### 2.2. Procedures

The variables used in this study were collected in three different manners by trained research staff and in accordance with the National Center for Education Statistics protocols. First, primary caregiver interviews were conducted by phone when possible. In instances when primary caregivers were difficult to reach by phone or preferred not to participate via phone, interviews were conducted in person. Second, child direct assessments were administered in schools where the children attended. Third, child indirect assessments were collected via teacher questionnaires, which were distributed and collected by trained research staff and were completed by participating teachers. For the current study, we used data collected in the spring of the kindergarten year (i.e., baseline data) for the selection of the analytic sample (i.e., NM-ACEs). To determine latent profiles of resilience, data collected in the spring of the first-grade year were used because child developmental functioning data were collected at this time point.

### 2.3. Measures

Variables included in the analysis fall into three categories. First, variables used to identify the analytic sample of children with NM-ACEs. Second, variables used to identify profiles of resilience. Third, family and student demographic variables that were used to explore demographic and ACE differences among the profile groups.

#### 2.3.1. NM-ACEs

Parental depression was taken from the parent respondents’ psychological well-being and health subscale of the parent interview. The primary caregiver was asked 12 questions from the *Center for Epidemiological Studies Depression Scale* [44], which is a widely used, well-validated measure for assessing depressive symptoms. Caregivers responded to questions such as “how often during the past week have you felt that you could not get going?”, using a 3-point response scale (i.e., never, some of the time, a moderate amount of time, or most of the time). The scale demonstrated high internal consistency (α = 0.86). To determine inclusion in the analytic sample, we first computed a composite score of the 12 items. Next, we examined the distribution and selected respondents in the quartile with the highest level of reported psychological distress. Respondents in the top quartile were then dummy coded to indicate parental depression (0 = no depressive symptoms, 1 = parental depressive symptoms).

The second adversity criterion considered was the poverty threshold. Poverty was assessed using a single item developed for the study that asked parents to disclose household income to interviewers. Study research staff used this information to determine if the family was below 100% of the poverty threshold, at 100% to below 200%, or 200% or above the poverty threshold for the community in which they lived. We dichotomized the poverty response to represent if a respondent was below 200% of the poverty threshold (=1).

Next, parental emotional or substance use problems were measured using a project-developed item. The question asks the respondent to respond yes (=1) or no (=0) to the prompt “during the past 12 months, have you felt or has anyone suggested that you needed professional help for any emotional problem or for drug or alcohol use?” Interviewers were supplied with text (e.g., unreasonable fears and inability to get along with others) to help respondents contextualize the question.

The final item we used as an indicator of NM-ACEs was whether the caregiver indicated that they had received benefits within the last 12 months. Using the project-developed questions, the ECLS-K team collected information from caregivers if they had received any of the following types of assistance: food assistance (food stamps), Temporary Assistance for Needy Families (TANF), and the supplemental nutrition program for women, infants, and children (WIC). We recoded these into a single variable to indicate if a family received any of these forms of assistance (=1) or not (=0).

#### 2.3.2. Profiles of Resilience

In order to determine profiles of resilience, we examined a variety of indicators from four domains of functioning: cognitive, executive, social, and behavioral function. All variables were collected in the spring of the first-grade year.

The cognitive domain was evaluated using *Item Response Theory (IRT)* scale scores for math and reading standardized assessments. Standardized reading and math assessments were administered in a two-stage assessment in order to determine ability and subsequently provide items appropriate for their ability level. We used the IRT scale score as it was designed to correct for any incomplete assessments based on the IRT-derived theta score, which measures a child’s functioning. Internal consistency was high for both measures (Reading α = 0.95; Math α = 0.94).

The executive function domain was assessed using two widely used, standard measures of cognitive flexibility and working memory. For cognitive flexibility, we used the *Dimensional Change Card Sort* [45]. The card sort is a task that asks children to sort 22 cards based on different rules such as color or shape. Two scores are produced based on the sorting processes: the post-switch score and the Border Game score. Following recommendations of the publisher and study authors [46], we used the combined score, which reflects the number of correct responses across the two sub-scores. For working memory, we used the *Numbers Reversed task* [47], which asks children to reverse a series of numbers that has been told to them. The task increases in complexity with the length of numbers. We used the Numbers Reversed W-ability score, which is a normed score constructed from data provided by the publisher, the number of correct items, the child’s age, and the language of the test the child was administered.

The social domain was assessed using teacher questionnaires that asked teachers to rate students on social skills and functioning. To assess prosocial skills, we used a measure of interpersonal social skills that is comprised of five items from the *Social Skills Rating System* [48]. The scale asks teachers to rate the frequency with which students demonstrate specific prosocial skills on a four-point scale from “Never” to “Very Often”. Item-specific data for the Social Skills Rating System are not available due to copyright restrictions. Second, the 15-item Student–Teacher Relationship Scale [49] was used to assess teacher closeness (e.g., this child spontaneously shares information about himself/herself) and conflict (e.g., this child and I always seem to be struggling with each other). Higher scores on the closeness subscale indicate a closer relationship a student has with the teacher. For teacher conflict, the score was reverse coded to aid in interpretability so that all outcomes (resilience functioning) would be interpreted with the same directionality. Higher scores on the teacher conflict scale indicated lower levels of conflict between teacher and student. Both subscales demonstrated a high level of internal consistency (prosocial: α = 0.86; teacher–child conflict = 0.89).

The behavioral domain was also assessed using established teacher questionnaires. To assess behavior outcomes, we used two subscales from the *Children’s Behavior Questionnaire* [50]. These subscales included six items to assess Attentional Focus and six items for Inhibitory Control. Higher scores on the Attentional Focus subscale correspond to an increased ability to focus attention on the current task. Likewise, higher scores on Inhibitory Control indicate better ability to resist the inclination to engage in inappropriate or off-task behaviors. Both subscales demonstrated a high level of internal consistency (Attentional Focus: α = 0.83; Inhibitory Control: α = 0.86).

### 2.4. Analysis Plan

The analytic sample was identified by children who had both complete data on the four NM-ACE measures and had at least one ACE, which indicated that they experienced some adversity. The outcome measures detailed above were used as indicators of the difference between resilience profiles. Because each scale contained different scoring, all outcomes were z-scored to aid in interpretability and make outcomes comparable to one another. Z-scores are interpreted such that the mean score is equal to zero and the standard deviation (SD) is equal to one. Thus, a child who scores 0.5 on an outcome is understood to be half an SD above the mean in that outcome.

To determine resilience profiles, the analytic sample was examined using a latent profile analysis (LPA) for 2 to 12 profiles using Mplus version 7.4 and following the procedure identified in Muthén and Muthén (2017) [51]. To aid in model fitting, we utilized Mplus LCA Helper [52], a free online tool that runs multiple models in sequence. Missing data were addressed in Mplus using full information maximum likelihood (FIML) estimation. To determine the best model fit, we extracted and compared model fit statistics including entropy, Lo–Mendell–Rubin likelihood ratio test (LMR), Parametric Bootstrapped Likelihood Ratio test (BLRT), and Bayesian Information Criterion (BIC). Lower BIC values and significant LMR and BLRT values were considered to indicate a better model fit [53]. In addition to these model fit indices, additional consideration was given to the interpretability and uniqueness of the identified profile [54]. Finally, we conducted one-way analysis of variance (ANOVA) and chi-square difference tests using SPSS 27 (IBM Corp, Armonk, NY, USA) to determine differences in demographic characteristics and adverse experiences across the identified profiles.

## 3. Results

### 3.1. Profile Identification

Full model fit results for models with two to eight profiles are presented in Table 2. We elected not to include fit statistics for models with nine to twelve profiles because of poor fit and redundancy. The final model we selected for examination was the four-profile model. The four-profile model was the best fitting model based on the examined fit statistics. First, BIC was reduced in the four-profile model by 1746 compared to the previous three-profile model. Second, the four-profile model demonstrated the highest entropy score of all the tested models (entropy = 0.881). Finally, the four-profile model was significant on both the Lo–Mendell–Rubin likelihood ratio test (*p* = 0.038) and the Parametric Bootstrapped Likelihood Ratio test (*p* < 0.001). To confirm that the four-model profile was the best fitting model, we graphed the two-, three-, four-, and five-profile models to compare them in terms of interpretability. Examining each model confirmed that the four-profile model was both the best fitting and most interpretable.

### 3.2. Profile Descriptions

**Figure 1:**
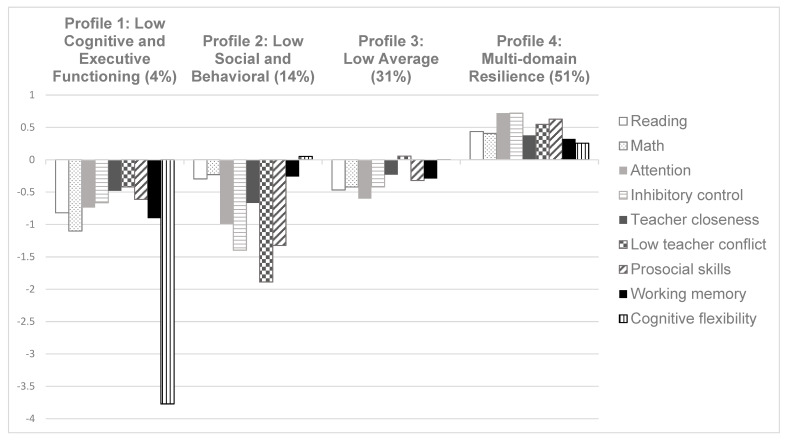
Latent Profiles of Resilience.

**Figure 2:**
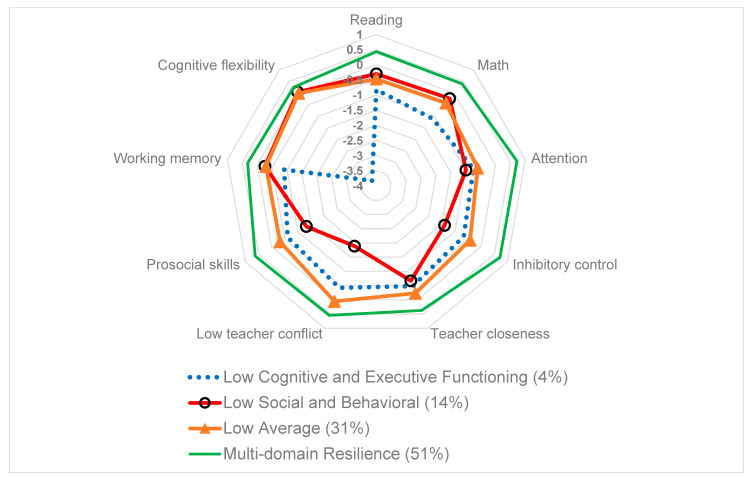
Radar Plot of Latent Profiles of Resilience.

Visual displays of the four profiles are provided in Figure 1 and Figure 2, with the same results shown in two different ways. The *low cognitive and executive functioning* profile represented 4% of the analytic sample (*n* = 181). These children were characterized by low resilience in cognitive and executive functioning outcomes. On average, these children were beyond a half standard deviation below the mean on all cognitive and executive function outcomes. Specifically, children in this profile scored on average −0.82 SD below the mean in reading and −1.1 SD below the mean in math. In executive function, children in this profile scored on average −0.9 SD below the mean on the numbers reversed task (working memory) and −3.77 SD below the mean on the Dimensional Change Card Sort (cognitive flexibility).

The *low social and behavioral functioning* profile made up 14% of the analytic sample (*n* = 721). This profile was characterized by children who scored more than half an SD below the mean on all social and behavioral resilience outcomes, but in comparison to the first profile were within 0.3 SD of the mean on cognitive and executive function outcomes. Specifically, children in this profile had lower behavioral resilience, –1.0 standard deviation below the mean in attentional focus and −1.4 SD below the mean in inhibitory control. On social resilience outcomes, children scored −1.33 SD below the mean on prosocial skills, −1.89 SD below the mean on teacher conflict (reverse scored), and −0.67 SD below the mean on teacher closeness.

The *low average functioning* profile represented 31% of the analytic sample (*n* = 1530). Children with the low average profile scored below the mean on all resilience outcomes, except for teacher conflict where students in this profile scored 0.06 SD above the mean. In all other resilience domains, children scored below the mean, including < 0.5 SD below the mean in six of the outcomes and −0.6 SD below the mean in attentional focus.

The *multi-domain resilience* profile was made up of children who scored above the mean on all measured outcomes. The multi-domain resilience profile represented 51% of the analytic sample (*n* = 2497). Across all measured resilience outcome domains, children with this profile scored between 0.25 and 0.75 SD above the mean.

### 3.3. Profile Demographic Differences

In order to determine if there were demographic characteristics and NM-ACEs that differed significantly between the four latent profiles, we conducted a series of one-way ANOVA and χ^2^ difference tests. Full results are presented in Table 3. The parents of the children with the *multi-domain resilience* profile were older (M = 35.2 years) than the parents of the children with the *low average functioning* profile (M = 34.5 years), *F* = 3.314, *p* = 0.019. Females were more likely to show *multi-domain resilience* compared to the other resilience profiles (χ^2^ = 85.49, *p* < 0.001). Children of races other than Black were significantly more likely to show the *multi-domain resilience* profile compared to the other resilience profiles (χ^2^ = 140.96, *p* < 0.001). Children who had parents with high school or more education were less likely to show the *low cognitive and executive functioning* profile and the *low average functioning* profile, compared to the *low social and behavioral functioning* profile or the *multi-domain resilience* profile (χ^2^ = 70.58, *p* < 0.001). Children whose household income was above 200% federal poverty level were more likely to show the *multi-domain resilience* profile compared to the other resilience profiles (χ^2^ = 77.28, *p* < 0.001). Similarly, children whose families did not receive benefits (e.g., food stamps, WIC, TANF) were more likely to show the *multi-domain resilience* profile compared to the other resilience profiles (χ^2^ = 112.77, *p* < 0.001).

## 4. Discussion

The primary aim of the study was to identify patterns of resilience in children exposed to NM-ACEs. Findings from this study contribute to a deeper understanding of heterogeneity in resilience functioning following exposure to NM-ACEs during kindergarten. As hypothesized, we found heterogeneous patterns of resilience functioning. Specifically, we found four distinct profiles of resilience: (1) *low cognitive and executive functioning*; (2) *low social and behavioral functioning;* (3) *low average functioning*; and (4) *multi-domain resilience*. The largest portion of the sample (51%) showed the *multi-domain resilience* profile in which children exhibited positive adaptation across a wide array of child functioning indices.

The finding of the *multi-domain resilience* profile containing the largest portion of the sample is consistent with previous studies that used a person-centered approach with children and youths exposed to adversities (e.g., foster care, exposure to violence) and reported a profile of positive adaptation and resilience to be the most prevalent profile. For example, Yates and Grey (2012) focused on emancipated foster youth and found almost half (47%) of the sample showing the resilient profile, which was characterized by youths faring reasonably well in all domains, including education, employment, civic engagement, and relational well-being [55]. Similarly, McDonald et al. (2016) examined patterns of adjustment among children exposed to intimate partner violence and found that the largest portion (66%) of the sample evidenced the resilient profile in which children demonstrated adaptive patterns of functioning across all measures, including empathy, social problems, attention problems, and internalizing and externalizing symptoms [56]. Together, these findings highlight the strength and resilience of children exposed to adversities, in line with the resilience framework [1,30,31].

The second largest profile was the *low average functioning* profile (31%) in which children scored below the mean on all indicators of resilience, except for teacher closeness. This profile is similar to moderate problems profiles identified in prior studies (e.g., the “struggling” profile in McDonald et al. (2016); the “maladapted” profile in Yates and Grey (2012) [55]). Though children with this profile showed low scores across the board, they did not exhibit clinically significant symptoms of psychopathology or severe impairment in any of the measured functions/domains.

The other two profiles, the *low social and behavioral functioning* profile (14%) and the *low cognitive and executive functioning* profile (4%), were smaller in size yet had distinct characteristics. These two profiles of resilience encompassed children who showed lower levels of resilience in certain domains of functioning. The *low social and behavioral functioning* profile was characterized by children struggling with behavioral adjustment and social relationships. This profile corroborates prior research that has documented higher levels of behavioral problems and low social competence among children exposed to a stressful family environment, including chaotic homes [14,15,57,58]. The *low cognitive and executive functioning* profile, while the smallest in size, represents one of the most interesting and novel findings of the study. This profile was characterized by lower cognitive functioning and executive functioning, especially cognitive flexibility, compared to the other profiles of resilience. A growing body of research suggests empirical evidence for impaired cognitive flexibility among children exposed to early life stress [59]. Yet, given its small size, this profile should be replicated and validated in future studies.

Our exploratory aim was to distinguish the differences in demographics and NM-ACE characteristics across the identified profiles of resilience. In terms of demographics, we found that older maternal age, child’s female gender, and races other than Black were associated with a higher likelihood of having the *multi-domain resilience* profile, compared to the other profiles of resilience. These findings are consistent with previous studies that found younger maternal age as a risk factor [60] and female gender as a protective factor [61] for resilience and positive child development. Yet, empirical results on the association between race and childhood resilience have been mixed and inconclusive, with some studies reporting better outcomes and higher resilience among White children [62] and other studies reporting no racial differences in childhood resilience [63]. More research is needed to understand the association between race and childhood resilience following exposure to adversity.

Children who had parents with high school or more education were less likely to show the *low cognitive and executive functioning* profile or the *low average functioning* profile. Interpreted within the broader child development literature, these findings corroborate past research that found significantly higher levels of cognitive functioning and executive functioning in children whose parents had a higher level of education, compared to children whose parents had a lower level of education [64,65]. Parents with higher educational attainment may be more involved in their children’s school education (e.g., discuss school activities/performance with the child, monitor the child’s use of time), which in turn may facilitate children’s cognitive development and academic achievement [66]. Furthermore, studies have found that parents’ higher cognitive abilities, such as intelligence quotient (IQ), are associated with better language and cognitive outcomes in children, suggesting the possible intergenerational transmission of cognitive abilities [67].

Notably, from the four NM-ACEs examined in this study, only household income and welfare benefits significantly distinguished between resilience profiles. Children whose household income was below 200% federal poverty level or received benefits (food stamps, WIC, TANF) were less likely to show the *multi-domain resilience* profile compared to the other resilience profiles. These poverty-related ACE findings, coupled with the parental education level findings, may suggest that family socioeconomic status (SES) plays an important role in children’s development of resilience.

### 4.1. Strengths and Limitations

The findings of the current study should be interpreted in light of the study’s limitations. First, many of the original NM-ACEs (e.g., imprisoned household members, parental divorce, domestic violence) were omitted or modified due to the lack of data availability. As with any secondary data analysis, this study was restricted to those variables and measures included in the dataset. For the same reason, maltreatment ACEs were not examined in this study. The findings should be replicated in future research with complete ACEs data. Second, we assessed NM-ACEs using parent interviews, which may be subject to social desirability bias. Given that parents were asked to disclose potentially sensitive and socially undesirable information, such as their emotional and substance abuse problems, the responses provided by the parents may not have accurately captured NM-ACEs. Third, due to the nature of the study design, no causal inferences can be made. Although we used data collected at two time points (demographic and NM-ACEs data collected at Time 1: the kindergarten year, outcomes data collected at Time 2: the first-grade year), child resilience outcomes data were collected only at Time 2, and thus we were unable to track changes in resilience profiles over time. Future research should consider examining resilience profiles longitudinally to see if children consistently show the same profiles of resilience or exhibit different resilience profiles over time.

Despite these limitations, the current study has unique strengths. The use of a nationally representative sample of kindergarteners/first graders allowed us to have greater confidence in the generalizability of the study results. Further, several methodological strengths are noteworthy, such as the large sample size, the use of sophisticated statistical techniques, the inclusion of a comprehensive array of child functioning measures as indicators of resilience, and the assessment of child functioning by multiple informants (i.e., parents, teachers). Finally, our application of the strengths-based theoretical framework and focus on childhood resilience contributes to the broader ACEs literature by moving beyond the traditional vulnerability/deficit model to understanding child outcomes in the context of ACEs.

### 4.2. Implications

Although our findings should be interpreted with caution in light of the aforementioned limitations, the study results provide several implications for practice, policy, and research. The identification of four distinct profiles of resilience in this study suggests that practitioners and clinicians working with school-age children with NM-ACEs should view these children as individuals with unique developmental strengths and needs. As such, it might be useful to consider a comprehensive assessment of child functioning across multiple domains, including cognitive and academic achievement, executive functioning, social relationships, and behavioral adjustment, to identify children’s unique developmental needs that could be targeted through interventions.

In terms of policy implications, based on our findings that the rate of the *multi-domain resilience* profile was significantly lower among children whose household income was below 200% federal poverty level or whose family received welfare benefits, there appears to be a need for increased funding to support positive child development for children in lower-income families. Additionally, the allocation of funds and resources to support household income (i.e., income support policies and initiatives) may be beneficial in helping children build resilience in the context of the stressful and disadvantaged family environment.

Within the research realm, our findings validate the importance of including NM-ACEs in considering the dose of childhood adversity that each person has experienced when studying resilience. Relatedly, future research should study other types of NM-ACEs, such as parental loss and parental incarceration, and incorporate these into the ACEs/trauma dose framework. Additionally, it may be beneficial to incorporate strengths-oriented measures, such as positive childhood experiences (PCEs) [68], to move beyond the risk-oriented ACEs measure and comprehensively assess childhood experiences and their relations to resilience profiles [69,70].

## 5. Conclusions

The findings from this study expand the current literature on resilience functioning among children and highlight the importance and utility of NM-ACEs as a critical construct in the field of resilience research. The identification of four distinct resilience profiles in this study provides empirical support for heterogeneity in resilience among children exposed to NM-ACEs and points to the need for conducting a comprehensive, multi-domain assessment of child functioning to promote optimal development in this population. Finally, the findings of this study call for more research on younger children who have experienced NM-ACEs and the need to follow these children over time to understand their various pathways to resilience functioning.

## Figures and Tables

**Table 1:** Sample Characteristics (*n* = 4929).

	*n*	%	*M* (*SD*)	Range
Child Characteristics				
Age at time of assessment (in years)	4904		7.2 (0.3)	5.8–8.8
Sex (female)	2477	50.3		
Race/Ethnicity				
White; Non-Hispanic	2168	44		
Black; Non-Hispanic	605	12.3		
Hispanic	1582	32		
Other races *	574	11.6		
Parent/Family Characteristics				
Age (in years)	4918		35 (7.5)	20–76
Race/Ethnicity				
White; Non-Hispanic	2372	48.1		
Black; Non-Hispanic	606	12.3		
Hispanic	1474	29.9		
Other	458	9.7		
Education level (less than high school)	906	18.4		
Household income (<200% federal poverty level)	3656	74.2		

Note. * Other races include Asian, Native Hawaiian/Pacific Islander, American Indian/Alaska Native, two or more races.

**Table 2:** Model Fit Indices for Resilience LPA Models.

Model	BIC	LMR	LMR *p*-Value	BLRT	BLRT *p*-Value	Entropy
2-class	114832	10357	<0.001	10479	<0.001	0.875
3-class	111606	3273	<0.001	3311	<0.001	0.864
**4-class**	**109860**	**1810**	**0.038**	**1831**	**<0.001**	**0.881**
5-class	108710	1220	0.635	1235	<0.001	0.814
6-class	107408	1371	0.271	1387	<0.001	0.827
7-class	106474	1007	0.160	1019	<0.001	0.844
8-class	105829	721	0.477	730	<0.001	0.834

Note. BIC = Bayesian Information Criterion; LMR = Lo–Mendell–Rubin Likelihood Ratio Test; BLRT = Parametric Bootstrapped Likelihood Ratio. Bolded class was selected as the best fitting class.

**Table 3:** Demographics and Non-maltreatment ACEs across Resilience Profiles (*n* = 4929).

	Latent Profiles				$\chi^{2}$	*F*	*p*	Post Hoc Differences
	Profile 1: Low Cognitive, Executive Functioning	Profile 2: Low Social, Behavioral	Profile 3: Low Average	Profile 4: Multi-Domain Resilience				
Maternal Age (years)	34.4	34.9	34.5	35.2		3.314	0.019	3 ≠ 4
Female (%)	49%	30%	43%	60%	85.49		<0.001	1,3 ≠ 2,4; 2 ≠ 4
Race/ethnicity								
White; Non-Hispanic	30%	45%	40%	47%	140.96		<0.001	1≠ 2,4; 2≠3
Black; Non-Hispanic	17%	22%	13%	9%				4 ≠ 1,3; 2 ≠ 3,4
Hispanic	40%	25%	36%	31%				2 ≠ 4; 1,3 ≠ 2,4
Other races *	12%	8%	11%	13%				2 ≠ 4
Parental education level (<high school)	23%	15%	25%	15%	70.58		<0.001	1,3 ≠ 2,4
Non-maltreatment ACEs								
Poverty (< 200% poverty level)	83%	78%	80%	69%	77.28		<0.001	4 ≠ 1,2,3
Received benefits	46%	49%	47%	33%	112.77		<0.001	4 ≠ 1,2,3
Parental depression	44%	47%	46%	50%	8.14		0.043	No sig diff
Parental emotional/substance use problems	12%	8%	8%	8%	4.94		0.176	No sig diff

Notes. ACEs *=* Adverse Childhood Experiences; * Other races include Asian, Native Hawaiian/Pacific Islander, American Indian/Alaska Native, two or more races.

## Data Availability

Publicly available datasets were analyzed in this study. These data can be found here: https://nces.ed.gov/ecls/dataproducts.asp (accessed on 16 June 2021).
